# Editorial: Aging, personal autonomy and independence

**DOI:** 10.3389/fragi.2023.1326657

**Published:** 2023-11-07

**Authors:** Alan Bruno Silva Vasconcelos, Pablo Jorge Marcos-Pardo, Marzo E. Da Silva-Grigoletto

**Affiliations:** ^1^ Department of Physiology, Federal University of Sergipe, São Cristóvão, Sergipe, Brazil; ^2^ CERNEP Research Centre, SPORT Research Group (CTS-1024), Department of Education, Faculty of Education Sciences, University of Almería, Almería, Spain; ^3^ Active Aging, Exercise and Health/HEALTHY-AGE Network, Consejo Superior de Deportes (CSD), Ministry of Culture and Sport of Spain, Madrid, Spain; ^4^ Department of Physical Education, Federal University of Sergipe, São Cristóvão, Sergipe, Brazil

**Keywords:** ageing, health, interventions, physical activity, psychology, multidomain interventions

The rise in the global population of older individuals, coupled with the surge in life expectancy observed in recent years, has prompted the World Health Organization (WHO) to declare the current era as the “Decade of Healthy Aging” (Amuthavalli Thiyagarajan et al., 2022). Programs operating within the framework of this initiative are not exclusive to the older population but encompass all demographic groups. Their primary goal is to guide individuals toward reaching an advanced age in optimal health, recognizing that health encompasses physical, psychological, and social dimensions.

As our global population continues to age, it becomes increasingly crucial to ensure that older individuals can age in a healthy and active manner. Chronic diseases represent significant health challenges as people advance in years (Maresova et al., 2019). Recognizing and enacting effective non-pharmacological preventive measures is a vital focus within the realm of public health, with many of these strategies requiring implementation in primary care settings. Presently, proven successful interventions involve comprehensive, multi-faceted programs that encompass strength, aerobic, flexibility, and balance training, cognitive exercises, nutritional education, and other domain-specific measures. There is a growing need to gain a deeper understanding of the most effective approaches to multi-dimensional interventions (Baker et al., 2007; Dedeyne et al., 2017; Trautwein et al., 2017; Whitney et al., 2019; Marcos Pardo et al., 2021).

The aging process contributes to the reduction of muscle mass, strength, and power (Keller and Engelhardt, 2013; Larsson et al., 2019). Consequently, functional fitness undergoes a gradual decline over the years, impairing the ability to perform activities of daily living safely, independently, and without excessive fatigue.

In view of the deleterious effects of aging, there is interest in developing and enabling therapeutic strategies that can attenuate these effects and, consequently, promote independence and quality of life in the older population. A focus on functional fitness variables, an important clinical outcome related to autonomy in older adults, has been recommended. Exercise, nutrition, and other health interventions appear to be effective in promoting multisystem adaptations in older adults.

The aim of this Research Topic was to increase knowledge about the interaction between different factors for the health of the older people. This compilation of studies provides a series of evidence on the efficacy of physical exercise interventions on the physical health, autonomy, and functional independence of the older people. It is necessary to continue working to educate from prevention at all ages to be able to age with health and have an active and healthy aging. This also involves educating all health professionals working in the community and in primary care centers to understand their role, and to work together with chartered physical activity educators to prevent, reduce and even reverse many chronic diseases that benefit from multidomain interventions.

Specifically, this Research Topic of articles adds to the limited body of literature focused on the consideration of aging, autonomy, and independence as a fundamental part of healthy multidomain physical activity programs.

Thus, this Research Topic covers Research Topic as varied as sleep apnea syndrome (SAS) in healthy Japanese older adults. Overall, this multifaceted study revealed that more than half of the study participants were at high risk for undiagnosed severe or moderate SAS (AHI >15/h) (Tanaka et al.). Another study investigated age-related changes in biceps femoris (BF) antagonist muscle coactivation during an acute recovery period following a leg extensor fatigue protocol (Harper and Thompson). In the following study we can learn that living alone was associated with a lower risk of decreased calf circumference among older adults, and could be a protective factor for sarcopenia (Wang and Zhang). In this study we can learn how a supervised intradialysis resistance training program for patients on brief daily hemodialysis treatment, as part of the clinical routine, can induce modest changes in body mass index, basal metabolic rate, and rise and fall time performance (Baião et al.). We will learn how features of the care environment can be used to facilitate the functional mobility of residents while reducing the risk of injury to staff in manual human handling interactions (MHP). And how the ProMob tool can be used to audit care facilities, plan for remodeling, and continuously improve care delivery and management of staff exposure to injury risk (Coman and Caponecchia). To conclude, this study suggests a bidirectional association between hand grip strength (HGS) and disability for activities of daily living (ADL). Low HGS can be used as a reliable marker of future disability for ADLs, which in turn exacerbates HGS decline. There is a need to strengthen screening and intervention of low HGS and improve functional recovery of individuals with ADL disability to promote healthy aging. In addition, it suggests how age and sex should be taken into account when assessing the association between HGS and ADL disability to develop more accurate and effective interventions (Dai et al.).

We hope you enjoy reading the articles included in this Research Topic and that you find them useful for your professional development in the pursuit of healthy ageing.
